# Research on Structural Performance of Hybrid Ferro Fiber Reinforced Concrete Slabs

**DOI:** 10.3390/ma15196748

**Published:** 2022-09-29

**Authors:** Hafiz Zain Saeed, Muhammad Zubair Saleem, Yie Sue Chua, Nikolai Ivanovich Vatin

**Affiliations:** 1 School of Engineering, Monash University, Jalan Lagoon Selatan, Bandar Sunway 47500, Malaysia; 2 Department of Civil Engineering, University of Engineering and Technology, Taxila 47080, Pakistan; 3 Peter the Great Saint Petersburg Polytechnic University, 195251 Saint Petersburg, Russia

**Keywords:** fiber-reinforced concrete slab, deflection, failure, stress-strain curve

## Abstract

Reinforced concrete structures, particularly in cold areas, experience early deterioration due to steel corrosion. Fiber-Reinforced Concrete (FRC) is an emerging construction material and cost-effective substitute for conventional concrete to enhance the durability and resistance against crack development. This article examines the structural performance of hybrid ferro fiber reinforced concrete slabs (mix ratio of mortar 1:2) comprising silica fume, layers of spot-welded mesh and different ratios of polypropylene fibers. The ferrocement slabs are compared with a conventional Reinforced Cement Concrete (RCC) slab (mix ratio of 1:2:4). The experimental work comprised a total of 13 one-way slabs, one control specimen and three groups of ferrocement slabs divided based on different percentages of Poly Propylene Fibers (PPF) corresponding to 0.10%, 0.30% and 0.50% dosage in each group. Furthermore, in each group, the percentage of steel ratio in ferrocement slabs varied between 25% and 100% of the steel area in the reinforced concrete control slab specimen. For evaluating the structural performance, the observation of deflection, stress-strain behavior, cracking load and energy absorption are critical parameters assessed using LVDTs and strain gauges. At the same time, the slabs were tested in flexure mode with third point loading. The experimental results showed that the first cracking load and ultimate deflection for fibrous specimens with 0.5% fiber and 10% silica fume increased by 15.25% and 13.2% compared with the reference RCC control slab. Therefore, by increasing the percentage of PPF and steel wire mesh reinforcement in the ferrocement slab, the post-cracking behavior in terms of deflection properties and energy absorption capacity was substantially enhanced compared to the RCC control slab.

## 1. Introduction

The construction industry is facing challenging civil engineering structures and ever-increasing material demands. Concrete contains about 10% by weight of cement [1]. Its performance is adversely affected due to micro-cracks formation before the application of loads, resulting in limited ductility, low tensile strength and little resistance against crack propagation [2,3]. Low tensile strength in concrete also leads to a brittle failure under the tension of about one-tenth of its compressive strength. Moreover, the manufacturing process of cement results in the emission of greenhouse gases; therefore, there is a need to develop and replace the conventional building construction material with an alternative environmentally friendly material [4,5]. To avoid higher costs from importing the materials, local materials are usually preferred for constructing floor and roofing systems in developing countries. The ductility of the concrete can be improved by adding fibers as a replacement for cement [6]. Ferrocement material, also known as thin reinforced concrete, is a versatile building construction material [7,8,9]. Due to comparatively better impact resistance, ductile performance, strength, and the readily casting feature, among other types of concrete, ferrocement material is considered an economical alternative for roofing systems [10]. It is a lightweight cementitious composite consisting of hydraulic cement mortar with closely spaced layers of continuous and relatively small size wire/weldmesh; cast to any shape due to its easy mouldability characteristic [11]. The recent applications of ferrocement in the structural engineering field include the construction of swimming pools, boats, silos, roofs, permanent formwork, underground water tanks, small deck bridges, strengthening and repairing of different reinforced structural elements, i.e., beams, slabs, columns, etc. In the last few decades, research has been done to understand its mechanical performance. The close spacing of the weld meshes in a cement-sand mortar improves the ductility and leads to a better crack arresting mechanism in ferrocement [12,13,14]. However, increasing the number of mesh layers beyond certain limits results in adverse effects like spalling matrix and delamination of extreme fibers; resulting in premature failure. Silica fume (SF) and fibers like aluminium, steel, polypropylene, have attracted much attention from the researchers worldwide [15,16]. Their addition to concrete is becoming common because they give excellent properties and improve flexural strength, toughness, ductility, impact strength, slippage and failure mode [17,18,19,20]. Adding or partially replacing SF with cement results in lower permeability and higher strength of concrete, whereas the addition of steel fiber enhances the tension capacity [21]. With the advancement in the construction industry, ferrocement nowadays is being used in different strengthening applications [22,23] besides its application for constructing thin building structural elements [24,25]. Therefore, more in-depth research is needed on this advanced material to improve its properties and the behavior of structural elements formed using ferrocement, particularly the flooring element, i.e., slab.

Mashrei et al. [26] examined the structural behavior of thirteen square ferrocement slabs (500 × 500 × 30 mm) under flexure mode of loading by varying the number of wire mesh layers, the presence of steel fibers (1%), and the percentage of SF (0% to 6%). Adding SF up to 4.5% and superplasticizers enhanced the strength of mortar in ferrocement slabs. The research concluded that the inclusion of 1% steel fibers increased the load-carrying capacity of slabs with and without SF by 29% and 18%, respectively. The ductility has also been enhanced by adding steel fibers, thereby reducing crack width and increasing the number of cracks. The load-carrying capacity has been enhanced up to 76% by increasing mesh layers from six to ten. Similarly, Shuxin et al. investigated the flexural behavior of FRP ferrocement plates with an ordinary composite of ferrocement having plain mortar and mesh [19]. The increase in tensile and flexural strength was reported to be 65–70% and 80% for the addition of PPF, 0.40% and 0.20%, respectively. The relationship between tensile and compressive strength has also been developed for self-compacting concrete with 80% cement replacement with industrial by-products and minerals [27]. 

The research by Murali et al. [28] examined the flexural and impact strength of 12 ferrocement slab panels under three-point flexural loading using expanded wire mesh (1,2 and 3 layers) and hooked steel fibers (1% and 2%). The investigated parameters were the first cracking load, ultimate load capacity, deflection, flexural strength, ductility index and crack width at ultimate and failure mode. The three layers of expanded wire mesh and 2% steel fibers significantly improved the ultimate load capacity, flexural strength, and ductility index of the ferrocement panels. The research showed that ferrocement panels could be used in many construction applications subjected to repeated low-velocity impacts.

Improvement in compressive and splitting tensile strength along with the durability of self-compacting concrete mixes was reported by many researchers [29,30,31]. Saleem and Ashraf carried out research on developing a low-cost, small house design [32]. They introduced panels of ferrocement as the main structural elements and a lightweight roofing system made up of a truss for additional resistance to earthquake forces. The seismic analysis was carried out on ETABS software having seismic zone 4, and the results were adequate under the seismic loading conditions. The application of ferrite in thin-walled structures was found ideally suitable because of the uniform dispersion and distribution of the reinforcing bars that provide better resistance to cracking, higher tensile strength, impact resistance and ductility. Adjusting the available mechanical production methods would be cost-effective in industrialized countries [33]. Flexural behavior of ferrocement slabs has also been investigated for load carrying capacity and deflection properties with different mesh layers. 

Ziadoon et al. investigated the flexural behavior of one-way ferrocement slabs with a thin mortar matrix under a four-point loading system. The main parameters of research were the number of mesh layers (1, 2, 3), types of fibers (golden steel fibers and waste aluminum fibers extracted from metallic can) and percentage of fibers (0.25, 0.5, 0.75) as per volumetric content. The results revealed that adding any type of fiber increases the ductility of tested slab more effectively than aluminum fibers. The increased number of wire meshes distributed along the thickness of the slab resulted in more strength capacity and ductile behavior [34]. The optimization of the reinforcement wire contentled to a general increase in the flexural strength, ductility, tensile strength and energy dissipation capabilities [35,36,37,38]. The experimental results also concluded that 0.6% of superplasticizer along with 20% fly ash and 5% SF resulted in improved flexural performance in strength and toughness [39]

Based on the above literature, sufficient information regarding the design and construction of various ferrocement structures [40,41,42] has been acquired with research and field experience. However, limited research is conducted on the flexural behavior of one-way ferrocement slabs reinforced with varying numbers of steel wire mesh and a binary combination of SF and PPF as additives. The present research aims to fill this gap and evaluate the structural performance of hybrid ferro fiber reinforced concrete slabs. The experimental program in current research is expected to present significant insights for implementing fibrous ferrocement panels subjected to a three-point bending test. The first cracking load, ductility, stress-strain relationship, and energy absorption capacity of hybrid ferro fiber reinforced concrete slab will be compared to conventional RCC slab. Therefore, the outcomes of this research will promote the application and extensive use of the hybrid ferro fiber reinforced concrete while solving the large material demands in the developing construction industry.

## 2. Materials and Methods

This experimental research program was designed to compare the flexural behavior of one-way ferrocement slabs with conventional reinforced concrete slabs as the control specimen. This section presents the properties of materials used, mix proportion, casting of specimens and flexure loading tests carried out.

### 2.1. Cement

Ordinary Portland cement (Type–I) from Bestway cement company in Pakistan was used for casting slabs as per the recommendations of ASTM C-150 [43]. Table 1 demonstrates the physical properties of cement used in this research.

### 2.2. Fine Aggregate

**Figure 1:**
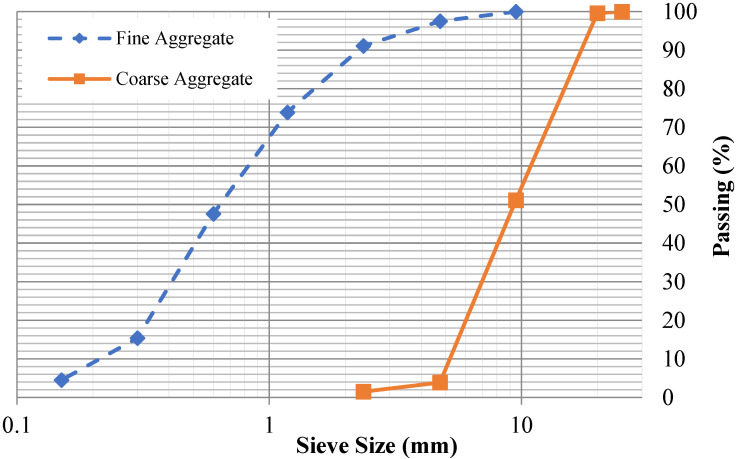
Particle size distribution of aggregates.

The quarry with the best fine aggregates is located in Lawrencepur, Pakistan. Sieve analysis test was performed in accordance with ASTM C136–05 [44] and the result is graphically shown in Figure 1. The fine aggregate (Lawrencepur sand) used in this research gave the fineness modulus of 2.70. Specific gravity and water absorption of fine aggregate (sand) were assessed in accordance with ASTM C 128-04a [45] and numerical values of 2.63 and 1.18% were obtained. 

### 2.3. Coarse Aggregate 

The quarry at Margalla in Pakistan was used as a source of coarse aggregates. A sample composed of 50% coarse aggregates passing through 37.5 mm (1.5 inches ) sieve and 50% passing a 20 mm (0.79 inch) sieve were mixed, followed by a sieve analysis test according to the recommendations of ASTM C136-06 [44]. The result is graphically shown in Figure 1. Specific gravity and coarse aggregate water absorption were assessed as per ASTM C 127-04 [46] and numerical values of 2.67 and 0.83% were obtained. The coarse aggregate′s unit weight/bulk density was also assessed as per ASTM C 29/C 29M-97 [47] and a numerical value of 1499.6 kg/m^3^ was obtained.

### 2.4. Water

The water used in the preparation and curing control and ferrocement slabs was clean from organic matter, sugar, oil, acids and chlorides, etc., and had a concentration of hydrogen ion on the PH scale as 7 was used.

### 2.5. Silica Fume (SF)

**Figure 2:**
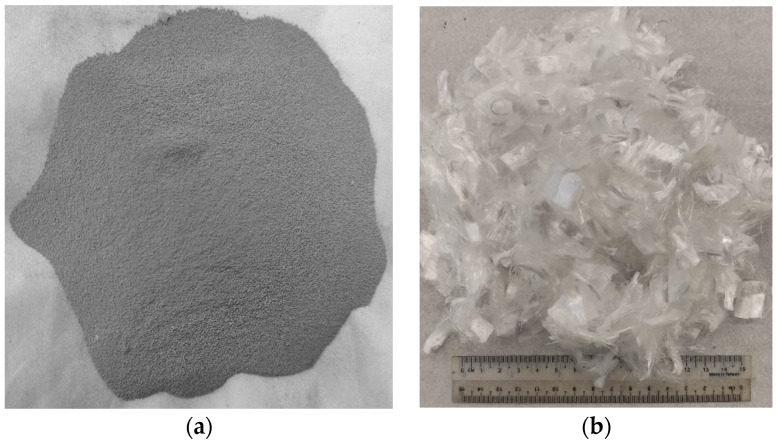
(**a**) Silica fume (SF) and (**b**) Polypropylene fiber (PPF) used in present research.

The silica fume (Figure 2a) used in this research was obtained from Imporient chemicals private limited (ICPL). It contains extremely fine, latently reactive silicon dioxide. The additional crystal formation produces a significantly denser cement matrix, resulting in high-strength, durable concrete. The physical and chemical properties of SF as binding material are tabulated in Table 2.

### 2.6. Polypropylene Fibers

The chemrite polypropylene fibers (Figure 2b) obtained from Imporient chemicals private limited (ICPL), Pakistan, was used in this research work. The detailed properties of PPF as a crack arrestor are tabulated in Table 3.

### 2.7. Steel Reinforcing Mesh

**Figure 3:**
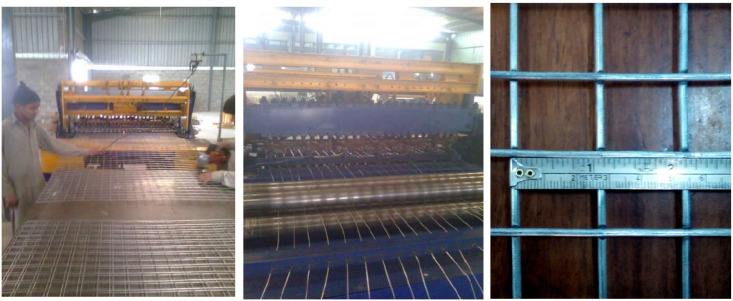
Preparation of spot-welded meshes.

Mild steel (MS) wires of imperial standard wire gauge (SWG) No.12, having a diameter of 2.64 mm (0.104 inches) spot welded together in a square mesh of 25.4 mm (1 inch) size, were used as reinforcement in ferrocement slabs. The different stages of making spot-welded wire meshes are shown in Figure 3. The bars were tested as per ACI 549.1R-93. The mean values for elastic limit, ultimate strength and elastic modulus of the welded steel wires are tabulated in Table 4.

### 2.8. Steel Reinforcement

The control specimen was cast using the deformed steel bars of size #3 having 9.5 mm (0.375 inches) diameter. Three specimens of these bars were tested under tension according to ASTM A615 [48] and reinforcement detailing was done per ACI 319 [49]. The yield and ultimate strength were recorded as 413.68 MPa (60,000 psi) and 586.05 MPa (85,000 psi) respectively.

### 2.9. Specimen Preparation 

**Figure 4:**
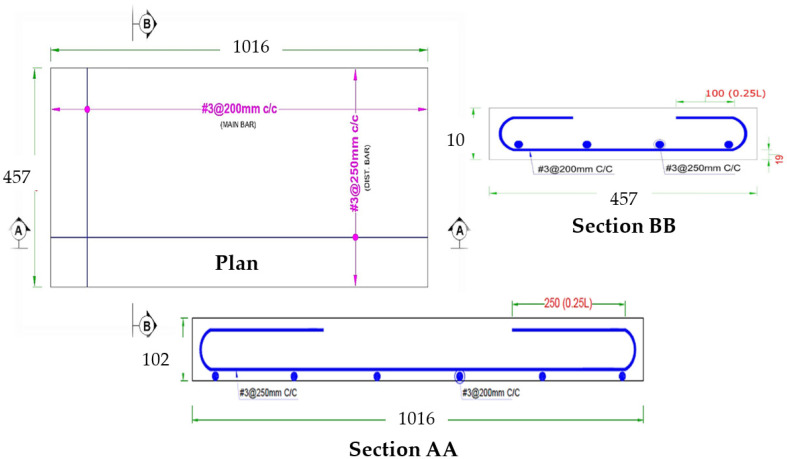
Reinforcement detail of control slab (All dimensions are in mm).

**Figure 5:**
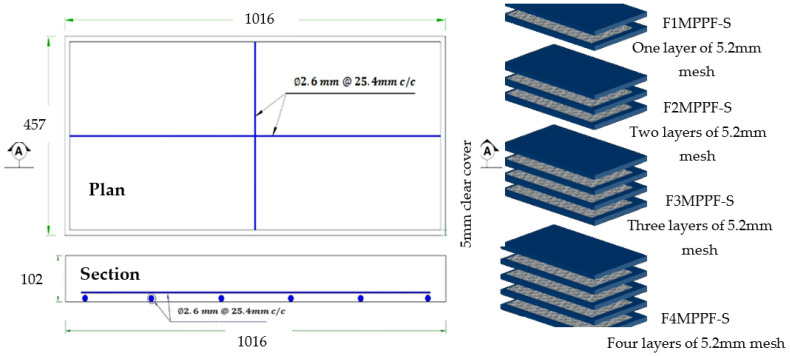
Reinforcement detail of ferrocement slab (all dimensions are in mm).

**Figure 6:**
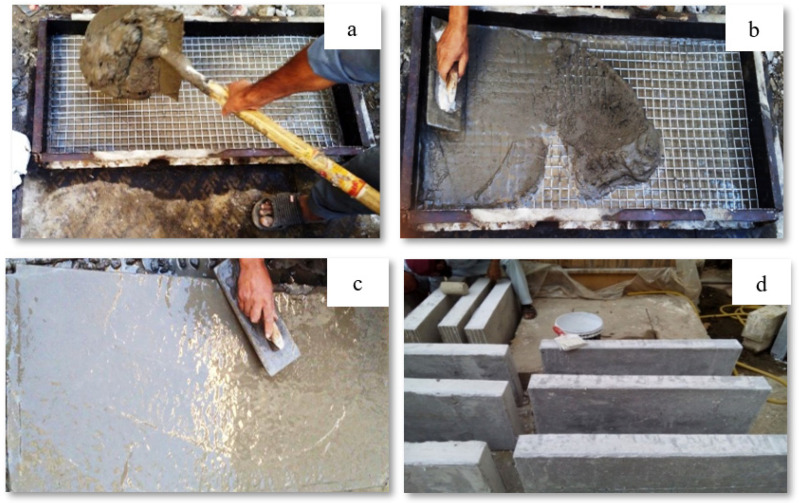
(**a**) Placing of ferrocement mesh and mortar; (**b**) Spreading of mortar over the mesh with a trowel; (**c**) Final finish with a trowel; (**d**) Whitewash on sides of slabs.

Past research evaluating the mechanical properties of mixes with partial replacement of cement with various industrial by-products suggested an optimum percentage of 15% for SF, 20% for fly ash and 0.5% for PPF [50,51,52]. ACI 549 [53] recommends a workable mix of mortar matrix with sand:cement of 1.4–2.5 and water:cement of 0.30–0.55 by weight of the matrix. The present research used a mix design of 1:2:0.50 (cement:sand:water) to cast ferrocement slabs of all groups tabulated in Table 5. The aim was to get the welded mesh fabric fully packed and densely coated with matrix while achieving maximum density, permeability and design strength. A total of 27 ferro-cement mortar cubes (9 for each of the three groups, i.e., A1, B1 and C1) and 9 reference concrete cubes with a mold size of 70.7 mm × 70.7 mm were cast and tested at a curing age of 3, 7 and 28 days as per the recommendation of the British Standard (BS 4550-3.4:1978) [54] and (BS-1881-116:1983) [55]. To evaluate the flexural performance, a three-point bending test was conducted. A total of 13 ferrocement slabs were divided into three groups, i.e., A1, B1 and C1 with 0.10%, 0.30% and 0.50% of PPF and 10% of SF, including one control slab. Furthermore, in each of the groups above, the percentage of steel ratio was varied (1, 2, 3 or 4 layers of 5.2 mm mesh) with an equivalent area of steel as the control slab. A mix ratio of 1:2:4 was adopted for the control slab and 1:2 for mortar used in ferrocement slabs. The slab specimen dimensions were 1016 mm (40 inches) in length × 457 mm (17.99 inches) in width × 102 mm (4.01 inches) in thickness for the 3-point bending test. All the steel molds were cleaned, oiled and tightened to prevent displacement when casting ferrocement and control slabs. Using glass spacer cover blocks, a clear cover of 3 mm (0.12 inches) was maintained in the formwork to cast ferrocement slabs. Firstly, sand, cement and SF were dry mixed. After that, 70% of water was added to the dry mortar matrix. The remaining 30% of water was added along with varying percentages of PPF. Finally, the mortar matrix was prepared, and mortar was placed into the formwork to cast ferrocement slabs. After 28 days of curing, all the ferrocement and control slabs were tested in flexure mode. Two coats of whitewash were applied on all four sides of the slab to clearly observe the crack patterns under flexural loading. The reinforcement details of the control and ferrocement slab are shown in Figure 4 and Figure 5. The detailed description of slabs cast in the experimental work and their designation are listed in Table 5 and graphically presented in Figure 6.

### 2.10. Experimental Procedure and Testing of Flexural Specimens 

**Figure 7:**
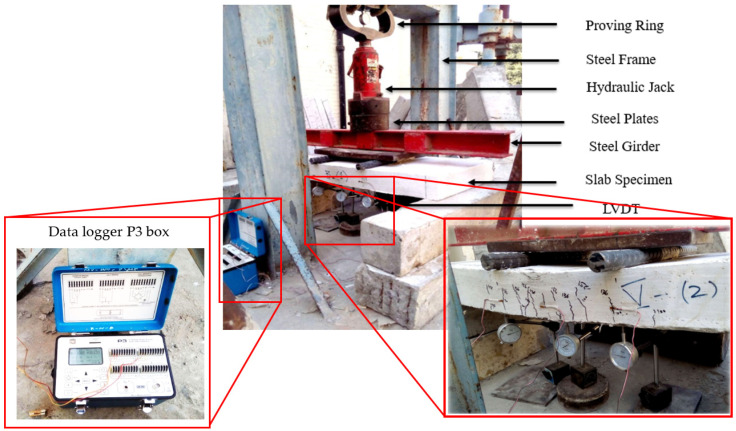
Testing setup for slab specimens.

This experimental study intends to evaluate the effect of SF incorporation along with PPF on the ferrocement slab behavior and mortar properties. For compression testing, a total of 36 mortar cubes were cast and tested at 3, 7 and 28 days of curing age. The results and discussion section presents the average values for the compressive strength of these cube specimens. A total of 12 ferrocement slab specimens and one control slab were cast and tested in flexure under 3-point loading as per ASTM C 78-02 [56]. The slabs were supported with a clear span of 1016 mm (40 inches). The experimental setup is shown in Figure 7. A proving ring of 3000 kN (674,426.83 lbf) capacity was used along with a hydraulic jack, steel plates and girders. The load was applied using a hydraulic jack in small increments; the mid-span deflection and strain of the center of the slab were recorded up to failure using linear variable displacement transducers (LVDT) and strain gauges. The increment of load in the proving ring was kept uniform (5 division interval) to observe the overall behavior of ferrocement and control slabs. The applied load and deflection were recorded in kilonewton (kN) and millimeters (mm). For every increment of load, the readings of LVDTs were carefully noted. The strain gauges were used to measure the strain response, and the data logger P-3 box recorded the data. After testing of slabs, their deflections and strains were noted. The following formula was used to calculate the flexural strength.

(1) $$\sigma =\frac{My}{I}$$

σ = Flexural stress (MPa), I = Moment of inertia = $\frac{bd^{3}}{12}$ (m^4^), M = Bending moment =$\;\frac{PL}{6}$,

Distance from Neutral axis = y = $\frac{d}{2}$ (m), P = Load (kN), b = Width (m), d = Thickness (m), L = Length of specimen (m).

## 3. Results and Discussions

### 3.1. Compressive Strength of Mortar Cubes 

**Figure 8:**
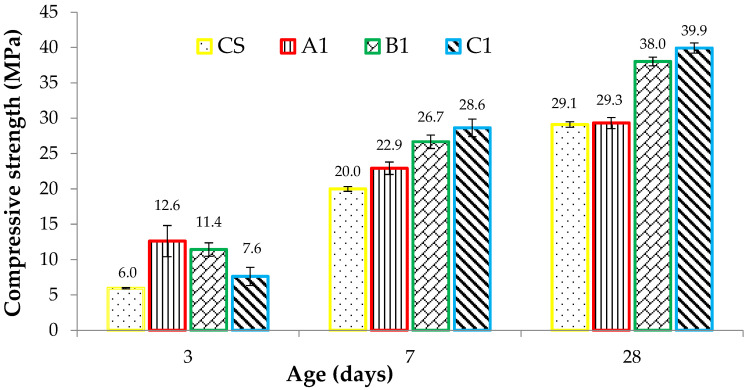
Compressive strength results of standard concrete and ferro-cement cube samples.

Compression test on cube specimens was performed to evaluate performance in a hardened state and summarized experimental results are graphically presented in Figure 8. X-ray diffraction (XRD) and scanning electron microscope (SEM) results in the literature showed that PPF does not participate in any chemical reaction [57]. Moreover, the PPF entrain small amounts of air during the surface treatment in their manufacturing process. Therefore, adding a higher percentage of PPF creates a more interfacial transition zone in concrete and results in a slight reduction of density and subsequently, the compressive strength [58]. This decreasing trend in compressive strength of ferro cement mortar cubes can be seen in Figure 8 at 3 days of curing age. However, the mechanical properties of fiber-reinforced concrete are affected by the distribution of fibers in the mixture, which can be enhanced significantly by adding SF, fly ash and slag [59,60]. It can be seen from Figure 8 that the incorporation of SF and increased percentages of PPF resulted in improved compressive strength of the mortar matrix at 7 and 28 days of curing age. The additionally added SF assisted in the dispersion of PPF, resulting in increased strength through improving the cement paste-aggregate bond [61,62]. Among the different cube specimens tested after 28 days of casting, ferro-cement cube (C1) with 0.5% PPF and 10% SF demonstrated the highest compressive strength of 39.9 MPa, which is 37.1% greater than the 29.1 MPa strength of control mix (CS) without PPF. The lower dosage of PPF (0.10% and 0.30%) also improved the 28 days compressive strength of CS cubes from 29.1 MPa to 29.3 MPa and 38.0 MPa, respectively. The comparatively higher compressive strength of ferro-cement cubes resulted from SF and increasing dosage of PPF fibers, effectively holding the micro-cracks propagation in concrete mass [63].

### 3.2. First Cracking and Ultimate Failure Load of Slab Specimens

**Figure 9:**
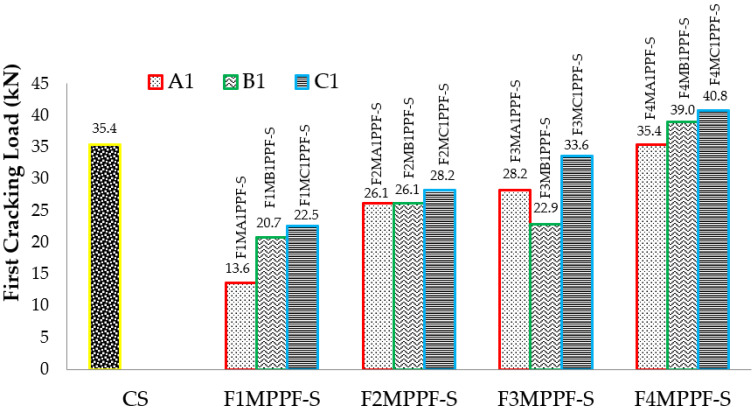
First cracking load results of ferrocement and RCC slab.

The load at which the first crack appears is called “First Cracking Load.” In control and ferrocement slabs, these cracks appeared in the center of the span and moved upward, showing pure flexural failure. Consistent with the material cube strength, the first cracking load of ferrocement slabs tested in flexure also demonstrated a comparatively higher load by adding PPF. Figure 9 shows the graphical representation of the first cracking load of different ferrocement slabs and control specimens. C1 group specimens endured the maximum value of the first cracking load with a maximum percentage of PPF. The specimen F4MC1PPF-S with 0.5% PPF and four layers of wire mesh, i.e., equivalent area of steel as that of the control slab (CS), showed a cracking load of 40.8 kN, which is 15.2% higher than the control slab (CS) with a first cracking load of 35.4 kN. This is due to an increase in specific surface area of mesh reinforcement, cumulative strength of steel meshes that were uniformly dispersed along the cross-section, and an increasing percentage of PPF [64]. The increased cracking behavior was evidently due to the contribution of PPF fibers, which acted as crack arrestors that helped to bridge the cracks increasing the first cracking load [65]. In group F3MB1PPF-S (Figure 9), corresponding to 0.30% of PPF and three layers of wire mesh (75% of steel area), the first cracking load is 22.9 kN which is even lower than F2MB1PPF-S with two mesh layer (25% of steel area) of the same group. This may be due to the premature failure caused by buckling/slippage of wire mesh in the mortar matrix.

**Figure 10:**
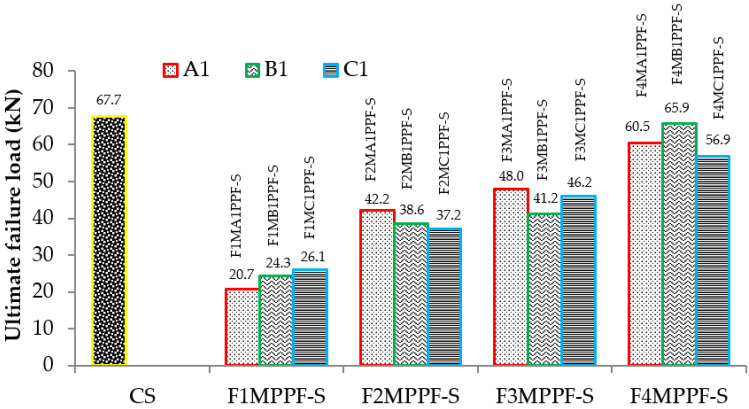
Ultimate load results of ferrocement & RCC slab.

Similarly, the results of ultimate load for varying percentages of PPF and wire meshes are graphically presented in Figure 10. This load in the tension zone is resisted by steel/welded wire mesh and in the compression zone by mortar/concrete alone or together with the tension zone. The load taken by mortar/concrete is limited; instantaneously, the cracks appear in the tension zone showing the transfer of stresses to the tensile zone. Among the various ferro fiber reinforced slab specimens, F4MB1PPF-S with 100% steel area equivalent to the controlled slab achieved the maximum value of the ultimate load. The ultimate load capacity in the F4MB1PPF-S ferrocement slab specimen with four layers of wire mesh increased by 171.2%, 70.7% and 60.0% compared to the corresponding one-layer (F1MB1PPF-S) two-layers (F2MB1PPF-S) and three -layers (F3MB1PPF-S) of wire mesh. The random orientation of PPF in the mortar matrix assists in controlling the propagation of cracks by enhancing the overall cracking resistance of the matrix and later by the arrest of micro-cracks formed just after the application of the load on the member. Therefore, preventing smaller cracks from expanding to major cracks results in higher ultimate load carrying capacity. It is worth mentioning that the enhancement in ultimate strength is due to an increase in fraction volume of the reinforcement in loading direction or increasing the numbers of wire mesh layers from 1 to 4 and is not influenced by their degree of dispersion.

### 3.3. Ductility Ratio

Ductility ratio is defined as the ratio of mid-span deflection at ultimate load to the mid-span deflection at first crack load. Ductile behavior is favored over brittle in design to minimize and avoid the loss of life and property. The principal component contributing to ductility is steel, i.e., rebar in the control slab and wire mesh in the ferro cement slab. The values of ductility ratio for varying percentages of polypropylene fiber and wire meshes are tabulated in Table 6. Consistent with the results from a previous study [66], improved resistance against crack growth is observed due to adding a lower dosage of PPF, resulting in a crack bridging effect and improved ductility. The ductility ratio for ferrocement slabs tested under flexure showed the highest value of 1.77 in the case of F4MB1PPF-S and the least value of 1.16 for F1MC1PPF-S. The trend is increasing by increasing the percentage volume fraction (V_r_) of reinforcement mesh [36]. Therefore, the presence of PPF inhibits intrinsic cracking in concrete. Thus, wire meshes and polypropylene fibers acted as crack arrestors and introduced significant ductility in the slab panel. 

### 3.4. Load Deflection Response

**Figure 11:**
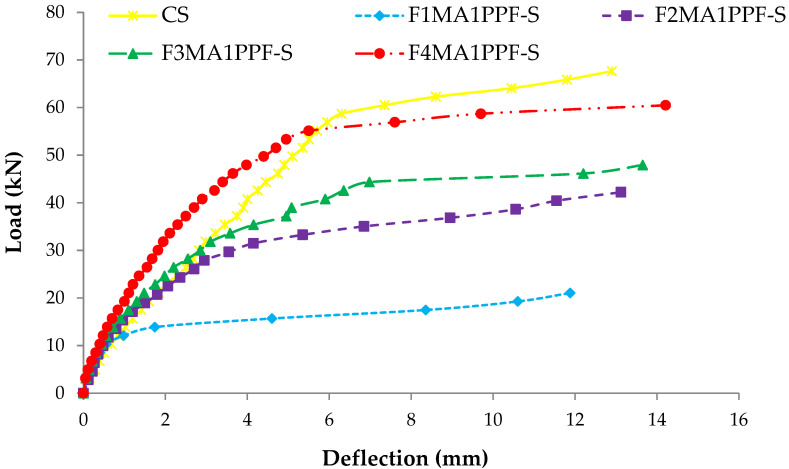
Comparison of load-deflection of group A1 with CS slab.

**Figure 12:**
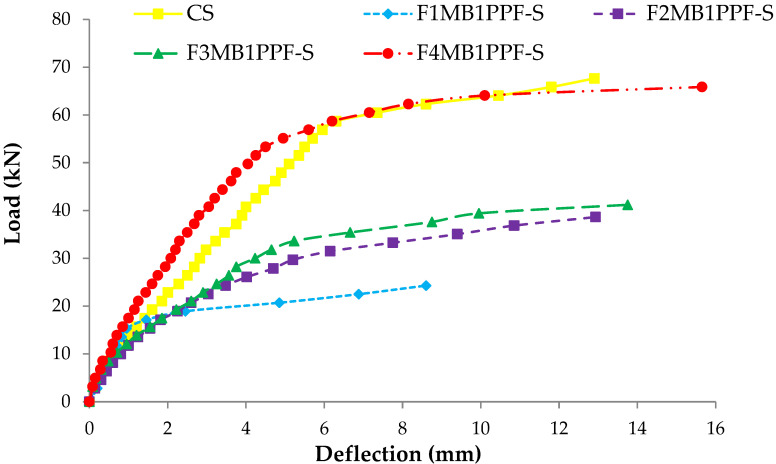
Comparison of load-deflection of group B1 with CS slab.

**Figure 13:**
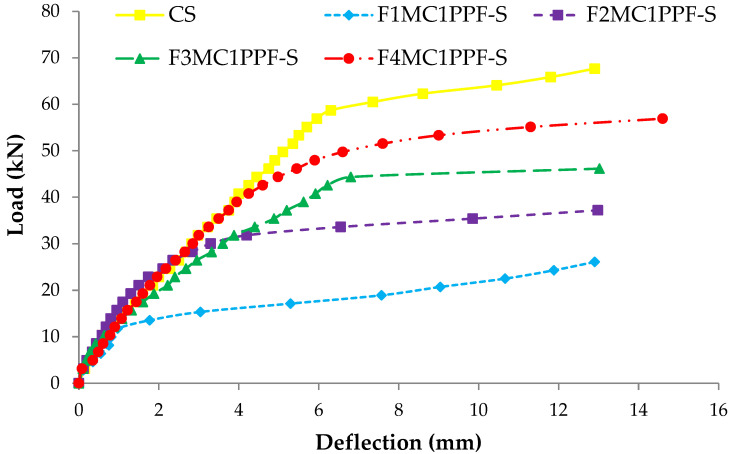
Comparison of load-deflection of group C1 with CS slab.

**Figure 14:**
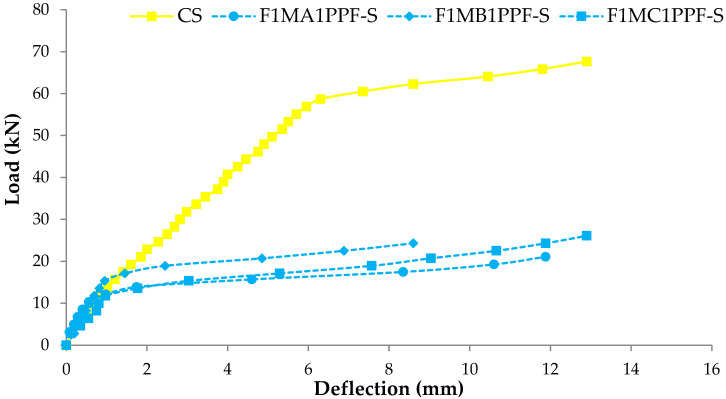
Comparison of load-deflection for different % of PPF and one steel wire mesh.

**Figure 15:**
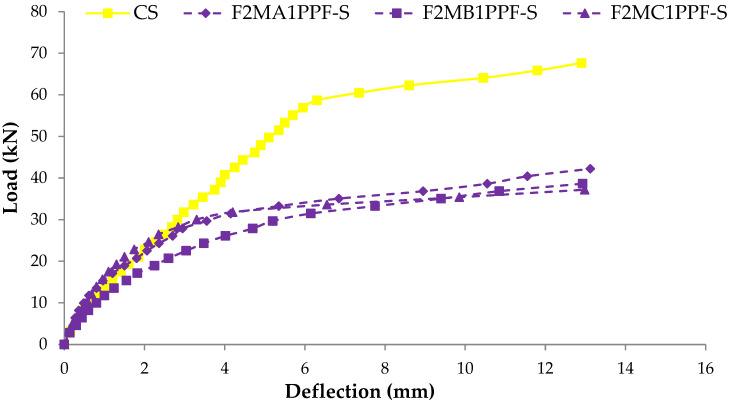
Comparison of load-deflection for different % of PPF and two steel wire mesh.

**Figure 16:**
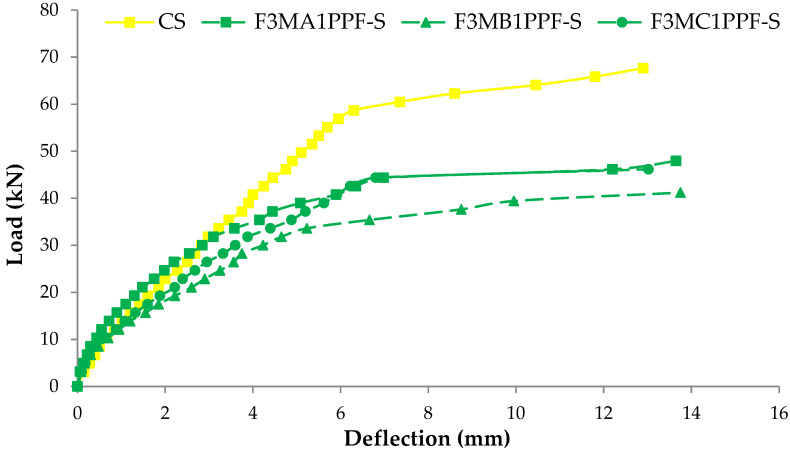
Comparison of load-deflection for different % of PPF and three steel wire mesh.

**Figure 17:**
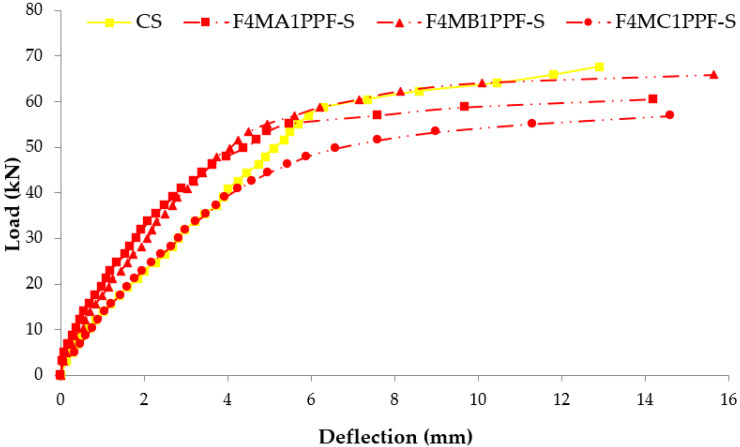
Comparison of load-deflection for different % of PPF and four steel wire mesh.

The comparison between load-deflection behaviors of ferrocement slabs with control slabs is plotted in Figure 11, Figure 12 and Figure 13. In contrast, the comparison of load-deflection for different percentages of PPF and varying numbers of steel wire mesh is presented in Figure 14, Figure 15, Figure 16 and Figure 17. All the load-deflection curves pass through elastic to failure stages [67]. As the reinforcement ratio increases in all the three groups i.e., A1, B1 and C1, there is a tremendous increase in load-carrying capacity and deflection among the ferrocement slabs. The deflection values of group A1 measured at failure points show an increased deflection behavior by 1.70%, 5.81% and 10.15% for mesh layers two, three and four, respectively. The maximum deflection of 11.9 mm is endured by F1MA1PPF-S, which is 7.96% less than the control slab deflection of 12.9 mm because of a lower percentage V_r_ of reinforcement, i.e., 0.429%. The deflection values of group B1 measured at failure points show an increased deflection behavior by 0.23%, 6.59% and 22.09% for mesh layers two, three and four, respectively. The deflection value measured at the failure point decreases behavior by 33.33% for mesh layer one, F1MB1PPF-S, compared to the control specimen. The reason is the slippage of steel mesh in the mortar matrix. F4MB1PPF-S contains maximum load-carrying capacity as compared to other groups A1 and C1 due to an excellent bonding effect of 0.30% of PPF with the cumulative effect of equal distribution of meshes along the cross-section; as a result, there is an increased behavior of deflection and strain as compared to mesh layer of other groups. The 0.30% of PPF tends to stretch more effectively in the face of crack separation. As a result, the load-carrying capacity is nearly equivalent to that of the control specimen. The deflection values of group C1 measured at failure points show an increased deflection behavior by 0.62%, 0.93% and 13.17% for mesh layers two, three and four, respectively. The load-carrying capacity of this group containing 0.50% of PPF is less than group A1 and B1 because higher percentages of PPF resulted in decreased cohesion and bond strength between mortar matrix and meshes [66]. The reason is that higher dosage rates are reducing the strength of the mortar matrix because higher volumes in fibers interfere with the cohesiveness of the mortar matrix.

### 3.5. Stress-Strain Response

**Figure 18:**
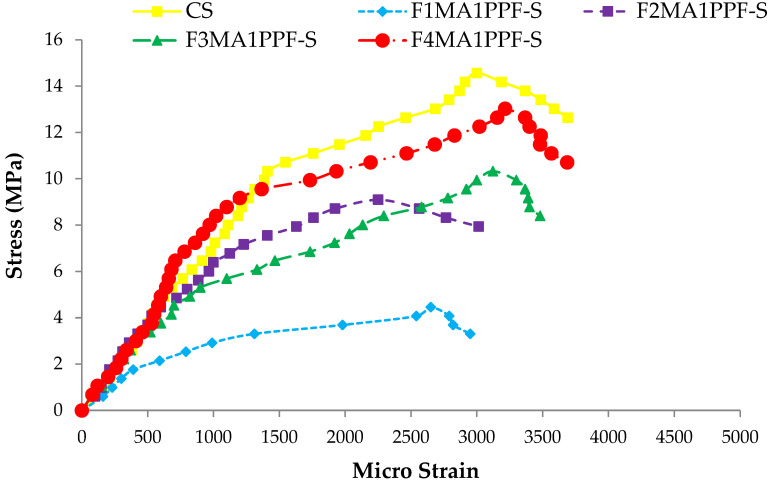
Comparison of flexure stress vs. strain of group A1 and CS slab.

**Figure 19:**
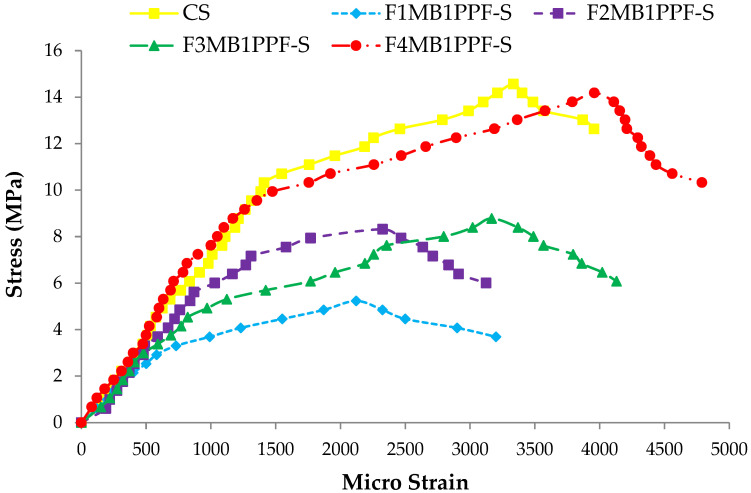
Comparison of flexure stress vs. strain of group B1 and CS slab.

**Figure 20:**
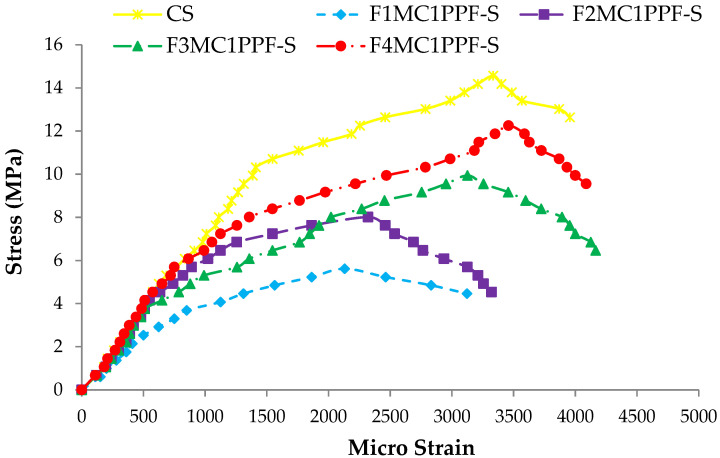
Comparison of flexure stress vs. strain of group C1 and CS slab.

The comparison between the stress-strain behavior of ferrocement slabs and control slabs (CS) is plotted in Figure 18, Figure 19 and Figure 20. The stress-strain curve represents a behavior similar to the load-deflection curve. The strain at maximum flexural stress for F1MA1PPF-S is 0.0027, which is 11.67% less than the control specimen due to a comparatively lower percentage of V_r_ for wire mesh reinforcement. Similarly, for F1MB1PPF-S, the strain at maximum flexure stress is 0.00212, which is 29.33% less than the control slab due to slippage of wire mesh in the mortar matrix. The strain noted at maximum flexural stress for F1MC1PPF-S is 0.00213, which is 29% less than the control specimen, mainly due to a lower percentage of V_r_ of reinforcement in the loading direction. 

While comparing the ferro fiber reinforced slabs with two wire mesh, the strain recorded at maximum flexural stress for F2MA1PPF-S is 0.00225, which is 24.97% less than the control specimen. On the other hand, the strain at maximum flexural stress for F2MB1PPF-S and F2MC1PPF-S is 0.0023, which is 22.50% less than the control slab. This anomalous behavior is due to the lower rate of strain increment with increasing load for mortar matrix due to the presence of PPF that acts to arrest the micro and macro cracks.

Similarly, comparing the ferro fiber reinforced slabs with three wire mesh, the strain at maximum flexural stress for F3MA1PPF-S is 0.00312, which is 4% more than the control specimen, i.e., the RCC slab. Moreover, the strain recorded at maximum flexural stress for F3MB1PPF-S and F3MC1PPF-S were 0.0032 and 0.0031, which compared to the control specimen are 6.67% and 3.33% higher. The steady increase in load with each strain increment is due to the combined effect of mobilization of the flexural capacity of the ferrocement slab and the role of PPF as a crack arrestor. 

For the last group of ferro fiber reinforced slabs with four wire mesh, the strain at maximum flexural stress for F4MA1PPF-S is 0.0032, which is 6.67% more than the strain of the control specimen. The steady increase in load with each strain increment is due to the uniform distribution of wire mesh reinforcement along the cross-section of the ferrocement slab specimen and the role of PPF in controlling intrinsic cracking. On the other hand, the strain at maximum flexural stress for F4MB1PPF-S is 0.0040, which is 33.33% more than the control specimen. The increase is due to the presence of 0.30% of PPF, which forms a network structure, leading to a reduction in stress concentration at the tip of the cracks and the role of %V_r_ of wire mesh reinforcement in the direction of load. The strain at maximum flexural stress for F4MC1PPF-S is 0.0033, which is 15.29% more than the control slab. The steady increase in load with each strain increment is due to the uniform distribution of wire mesh %V_r_ along the cross-section of ferrocement slab specimen and the role of 0.50% of PPF to control intrinsic cracking. The increase in stress at any particular deformation is due to the presence of PPF, which forms a grid structure resulting in lowering the stress concentration at the crack’s tip and the contribution of fraction volume of reinforcement in the direction of load.

### 3.6. Energy Absorbed

**Figure 21:**
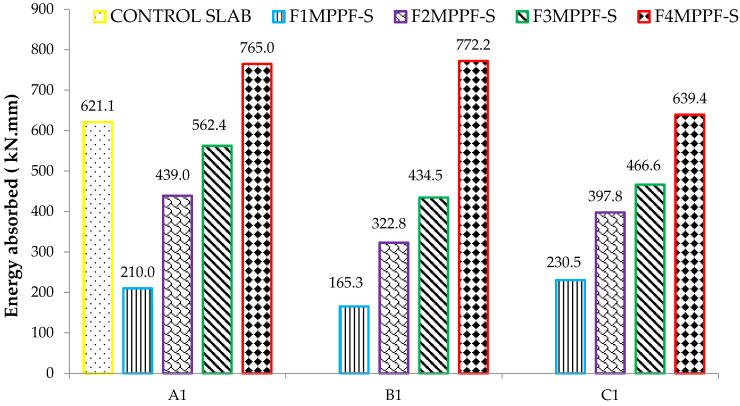
Absorbed energy of ferrocement and control slab.

Energy absorption is a characteristic feature of ferro fiber reinforced composites during their deflection hardening stage. The absorption energy is determined by calculating the entire area under the load-deflection curve from origin to failure, which indicates the toughness of deflection at mid-span [53]. MATLAB software determined the energy absorbed by finding the area under the load-deflection curves. The results of absorbed energy by ferrocement and control slabs are presented in Figure 21. The comparison between the control slab and the ferrocement slab (four layers of wire mesh) shows a significant increase in energy absorption values, i.e., 23.16%, 24.16% and 2.95%, corresponding to 0.10%, 0.30% and 0.50% of PPF, respectively. The increase in the energy absorption capacity of slabs with 0.10% and 0.30% of PPF is more than the slab with 0.50% of PPF due to the excellent bonding effect of fibers with mortar matrix. The higher dosage rate in group C1 with 0.5% of PPF is responsible for reducing the strength of the mortar matrix as higher volumes of fibers interfere with the cohesiveness of the mortar matrix.

### 3.7. Crack Pattern

**Figure 22:**
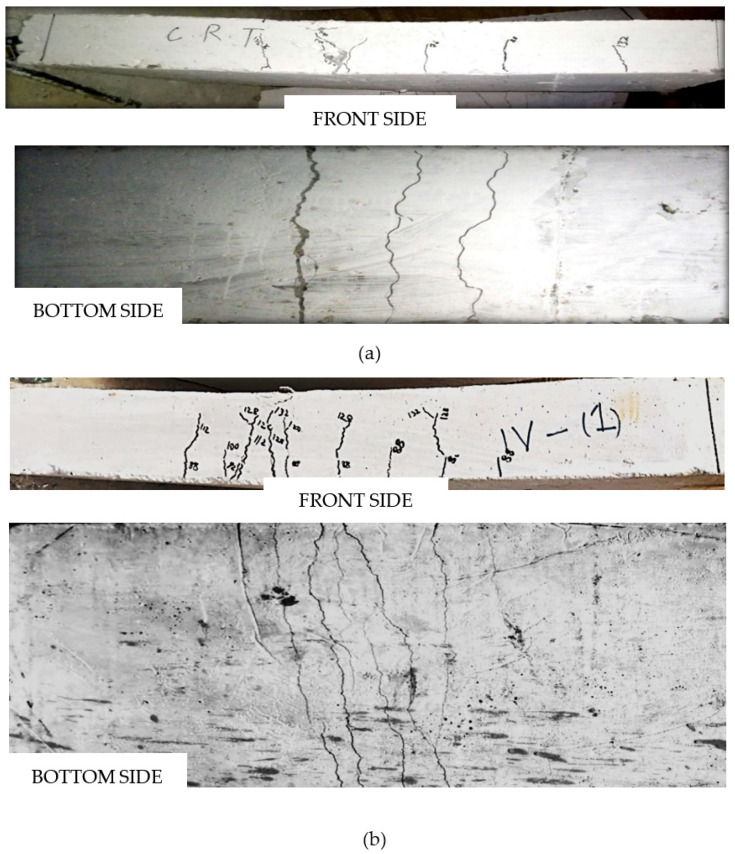

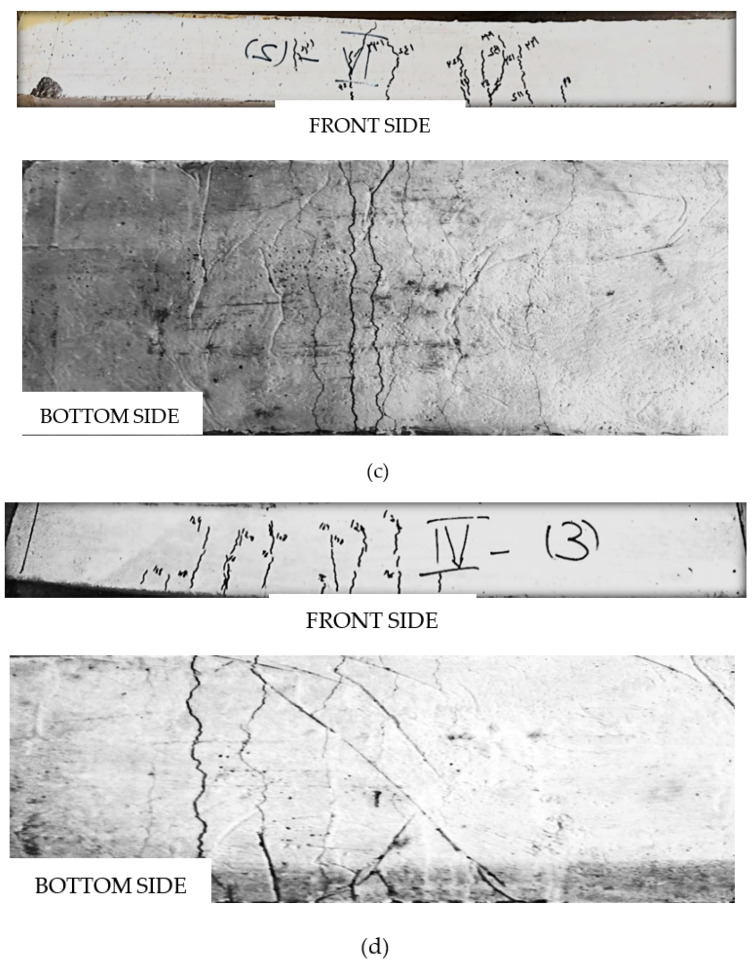
Typical crack pattern of control slab and ferrocement slabs with equivalent steel area (four steel wire mesh). (**a**) Crack pattern of CS, concrete control slab. (**b**) Crack pattern of F4A1MPPF-S slab (0.1% PPF), 0.1% of PPF percentage. (**c**) Crack pattern of F4B1MPPF-S slab (0.3% PPF), 0.3% of PPF percentage. (**d**) Crack pattern of F4C1MPPF-S slab (0.5% PPF), 0.5% of PPF percentage.

Flexural failure mode was observed for all the tested slab specimens, i.e., yielding of the steel wires with no indication of mortar crushing at the compression face of the cross sections. The crack patterns of control and ferrocement slabs with equivalent steel area (four wire mesh) are shown in Figure 22. The control slab comprised conventional concrete and had a somewhat indefinite profile of crack patterns. The reason was the difference in resistive strength in compression and tension zone of slabs. All the cracks started from tension to the compression zone. While observing the bending behavior of all the ferrocement slabs, the first crack pattern appeared just below the load application point. More cracks appeared after the first crack on either side of the first crack when the load increased at higher levels [12]. In the tension zone, the crack in the mortar matrix occurs as polypropylene fiber starts acting. The fiber carries the load across the crack, transferring the load from one side of the lattice. As the fiber is randomly distributed, the cracks do not have very long paths, thereby increasing load bearing capacity of the whole mortar matrix [68]. The presence of PPF in the ferrocement slab resulted in multiple cracks with a comparatively smaller width than the control slab specimen.

## 4. Conclusions

The present research achieved higher compressive strength due to the incorporation of 10% SF. This helps to improve the cement paste-aggregate bond and distribution of PPF. The ductility loss was compensated by adding a different percentages of PPF and multiple layers of wire mesh in ferrocement slabs, resulting in a higher specific surface of reinforcement and the development of larger bond forces. The research verifies that ferrocement slabs are superior in controlling cracks and have a higher cracking load than similar control slabs with normal concrete. The following conclusions are drawn from the present research:

1. Replacement of cement with 10% SF and adding PPF in cubes enhanced the compressive strength, with the maximum percentage recorded as 37.11% with 0.50% polypropylene fibers. SF improved the dispersion of PPF and increased the compressive strength through an improved bond between cement paste and aggregates.
2. First cracking and ultimate loads depended on the number of wire mesh layers in ferrocement slabs.
3. Higher numbers of wire meshes and their uniform distribution along the thickness of ferro cement slabs resulted in more ductility. This resulted in more cracks with reduced width.
4. A comparative study of the first cracking load showed that adding PPF and reinforcement optimization in ferrocement slabs (four meshes, 0.50% PPF and 10% of SF) resulted in 15.25% higher values than the control slab.
5. Increasing strain at peak stress and extended length of descending branch for the stress-strain curve was observed with an increased percentage of polypropylene fibers in the mixture.
6. A similar increasing trend of 24.33% was observed for the energy absorption capacity of ferrocement slabs (four meshes, 0.30% PPF and 10% SF), proving enhanced ductile behavior and superiority in crack control for ferrocement slabs.
7. A comparative study of deflection behavior showed that ferrocement slabs (four meshes, 0.30% PPF and 10% of SF) experienced a maximum of 13.56% more deflection than the control slab, exhibiting good ductility. Similarly, the percentage increase in strain observed with ferrocement slabs was 21.05% more than that of the control slab.
8. The ductility ratio for ferrocement slabs showed the highest value of 1.77 in the case of F4MB1PPF-S and the least value of 1.16 for F1MC1PPF-S. The increasing trend was due to the increased % V_r_ of reinforcement mesh.

Following recommendations for future work are suggested by the authors.

1. Further research can be done to study the influence of mesh opening, mortar layer thickness and section depth on the flexural behavior of ferrocement slabs.
2. More experimental work is required to study the flexural behavior of reinforced ferrocement slabs with other mesh types. The most economical type of mesh optimization study can then be carried out. Moreover, the durability aspects of selecting a curing technique and its optimum duration must be explored.
3. Further experimental works are needed to explore the feasibility of using recycled aggregates instead of natural coarse aggregate concrete.
4. SEM studies need to be conducted to better understand the binary action of SF and PPF on the concrete internal pores filling mechanism.
5. Finite element analysis (FEA) and parametric study for investigating the effects of concrete strength and cover on the load carrying capacity and ductility performance of hybrid ferro fiber reinforced concrete slabs.

## Figures and Tables

**Table 1:** Properties of ordinary Portland cement (OPC).

Parameters	Numerical Values
Consistency	29.34%
Soundness	No expansion is seen.
28 days compressive strength	40.88 MPa
Specific gravity	3.06
Initial setting time	1 h, 53 min
Final setting time	3 h, 56 min

**Table 2:** Physical properties and chemical composition of silica fume.

Parameters	Numerical Values	Chemical Composition	Percentage (%)
Appearance	Grey powder	SiO_2_	90–95
Amorphous	SiO_2_ > 90%	Al_2_O_3_	4
Specific gravity	2.2–2.3	Fe_2_O_3_	5
Specific surface	15–30 m^2^/g	MgO	5
Mean particle size	0.5 μm	CaO	3
Dry bulk density (Avg)	450 Kg/m^3^	-	-

**Table 3:** Properties of polypropylene fibers.

Parameters	Numerical Values
Form	White fibers
Density	0.9 ± 0.01 kg/L
Fiber diameter	15–30 micron
Tensile strength	300–450 MPa
Softening point	160 °C
Alkali resistance	100%
Specific surface area	Approx. 200 m^2^/kg
Thermal conductivity	Low
Chop length	6, 9, 12 & 19

**Table 4:** Properties of steel wire mesh.

Property of SWG MS Mesh	Numerical Values
Diameter	2.6 mm
Opening of mesh	25.4 × 25.4 mm
Type	Square-spot welded
Weight	3.273 kg/m^2^
SWG gauge	12
Volume	4.549 × 10^−4^ m^3^/m^2^
Yield strength	224 MPa
Ultimate strength	294 MPa

**Table 5:** Details of ferrocement slabs cast according to the mix design.

**Sr. No**	**Specimen** **Designation**	**Group**	**No. of Steel Wire Mesh**	**Area of** **Steel (%)**	**Mix Ratio**	W/binder ** **Ratio**	**PPF** **(%)**	**SF** **(%)**	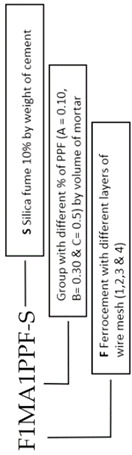
1	RCC	Control Slab *	--	100	1:2:4	0.50	--	--	
2	F1MA1PPF-S	A1	1	25	1:2	0.50	0.10	10	
3	F2MA1PPF-S		2	50	1:2	0.50	0.10	10	
4	F3MA1PPF-S		3	75	1:2	0.50	0.10	10	
5	F4MA1PPF-S		4	100	1:2	0.50	0.10	10	
6	F1MB1PPF-S	B1	1	25	1:2	0.50	0.30	10	
7	F2MB1PPF-S		2	50	1:2	0.50	0.30	10	
8	F3MB1PPF-S		3	75	1:2	0.50	0.30	10	
9	F4MB1PPF-S		4	100	1:2	0.50	0.30	10	
10	F1MC1PPF-S	C1	1	25	1:2	0.50	0.50	10	
11	F2MC1PPF-S		2	50	1:2	0.50	0.50	10	
12	F3MC1PPF-S		3	75	1:2	0.50	0.50	10	
13	F4MC1PPF-S		4	100	1:2	0.50	0.50	10	

* The W/C ratio of 0.50 is used for control specimen ** Binder = Cement + SF.

**Table 6:** Ductility ratio of ferrocement and controlled slabs.

Sr. No	Type of Slab	Percentage of PPF (%)	1st Cracking Load in Flexure (A)	Ultimate Load in Flexure (B)	Ductility Ratio (B/A)
1	Control	NIL	35.41	67.66	1.91
2	F1MA1PPF-S	0.1	13.55	20.72	1.53
	F1MB1PPF-S	0.3	20.72	24.31	1.17
	F1MC1PPF-S	0.5	22.52	26.10	1.16
3	F2MA1PPF-S	0.1	26.10	42.22	1.62
	F2MB1PPF-S	0.3	26.10	38.64	1.48
	F2MC1PPF-S	0.5	28.24	37.19	1.32
4	F3MA1PPF-S	0.1	28.24	47.95	1.70
	F3MB1PPF-S	0.3	26.45	41.22	1.56
	F3MC1PPF-S	0.5	33.62	46.15	1.37
5	F4MA1PPF-S	0.1	35.41	60.49	1.71
	F4MB1PPF-S	0.3	37.18	65.86	1.77
	F4MC1PPF-S	0.5	40.78	56.91	1.40

## Data Availability

All the data is available within this manuscript.
